# PromptSeg: An End-to-End Universal Medical Image Segmentation Method via Visual Prompts †

**DOI:** 10.3390/e28030342

**Published:** 2026-03-18

**Authors:** Minfan Zhao, Bingxun Wang, Jun Shi, Hong An

**Affiliations:** School of Computer Science and Technology, University of Science and Technology of China, Hefei 230026, China; zmf@mail.ustc.edu.cn (M.Z.);

**Keywords:** medical image segmentation, in-context learning, universal segmentation

## Abstract

Deep learning has achieved remarkable advancements in medical image segmentation, yet its generalization capability across unseen tasks remains a significant challenge. The variety of task objectives, disease-dependent labeling variations, and multi-center data contribute to the high uncertainty of task-specific models on unseen distributions. In this study, we propose PromptSeg, an innovative Transformer-based unified framework for universal 2D medical image segmentation. From an information-theoretic perspective, PromptSeg formulates the segmentation process as a conditional entropy minimization problem, utilizing visual prompts as side information to reduce the uncertainty of the target task. Guided by the information bottleneck principle, PromptSeg aims to utilize the provided visual prompts to filter out redundant noise and learn contextual representations, thereby breaking the restrictions of the task-specific paradigm. When faced with unseen datasets or segmentation targets, our method only requires a few annotated visual prompt pairs to extract task-specific semantics and segment the query images without retraining. Extensive experiments on CT and MRI datasets demonstrate that PromptSeg not only outperforms state-of-the-art methods but also exhibits strong multi-modality generalization capabilities.

## 1. Introduction

Precise segmentation of anatomical structures in medical images is pivotal for clinical diagnosis, treatment planning, and image-guided interventions. Over the past decade, deep learning approaches have revolutionized this field, achieving expert-level performance on specific tasks [1,2,3,4,5,6]. However, the standard paradigm—training specialized neural networks for specific organs or datasets—encounters significant bottlenecks in clinical deployment [7]. First, these **task-specific** models lack flexibility; the segmentation performance significantly degrades when applied to unseen anatomical targets or distinct imaging modalities due to the domain shift. From an information processing standpoint, this failure stems from the high uncertainty inherent in unseen data distributions, which fixed-weight models cannot resolve without additional contextual information. On the other hand, training a segmentation model from scratch for a specific task typically requires large-scale annotated data, leading to high costs.

To address these issues, considerable efforts have been devoted to exploring methods such as transfer learning and few-shot learning. Transfer learning strategies aim to adapt pre-trained models to new tasks through fine-tuning, thereby reducing the demand for annotated data [8,9,10]. Some studies have transferred pre-trained models obtained from large-scale natural image datasets [10,11,12,13], such as ImageNet [14] and COCO [15], to medical image segmentation tasks. While effective to some extent, the substantial feature discrepancy between natural and medical scenes often limits the efficacy of feature reuse [12]. Few-shot-learning-based methods aim to segment query images by leveraging a “support set” containing a few annotated examples. Classic few-shot learning approaches often employ a “learn-to-learn” strategy. These methods require designing a specialized model to construct a representative prototype from the training set and then obtaining segmentation results by matching support and query representations [16,17,18]. Although these methods demonstrate rapid adaptation, they often struggle with cross-modality generalization and fail to capture complex structural variations outside their training domains. Recently, UniverSeg [19] introduced a task-agnostic framework trained on a large-scale and diverse annotated dataset, showing promise in universal segmentation. UniverSeg demonstrates strong generalization ability on unseen anatomical structures and tasks, outperforming existing few-shot methods. However, its reliance on lightweight convolutional blocks restricts its capacity for high-precision segmentation and model scalability.

In recent years, the field of natural language processing (NLP) has witnessed a paradigm shift with the advent of “prompt learning,” which enables the model to infer task objectives from input prompts and adaptively learn from context [20,21]. Existing large language models (LLMs), such as GPT-4, adopt the prompt learning strategy to enhance the generalization ability across various language tasks without parameter updates. Inspired by this success, the computer vision community has begun to investigate prompt learning for natural image analysis such as inpainting and segmentation [22,23]. These methods are no longer limited to specific task objectives but can perform well on various tasks such as image classification, reconstruction, and segmentation defined by the provided visual prompts. However, most existing methods are designed for general image analysis tasks rather than just the segmentation task and rely on complex proxy tasks and training procedures.

To overcome the above problems, we propose an end-to-end universal medical image segmentation method based on prompt learning, named **PromptSeg**. The core idea is to utilize visual prompts to guide the model in segmenting query images for specified targets, without being limited to specific tasks and data modalities. We interpret the visual prompting process as a conditional entropy minimization mechanism, where the prompts serve as side information to maximize the mutual information between the input context and the prediction. Our main contributions can be summarized as follows:

- We introduce prompt learning into medical image segmentation, creating a novel end-to-end universal segmentation method.
- We propose PromptSeg, a task-agnostic framework that employs an image-level auto-regressive model to segment query images through next-image prediction, using a handful of image–mask pairs with the same segmentation class as visual prompts to indicate the task. Furthermore, we aggregate existing open-source datasets to construct a large-scale, multi-source medical segmentation dataset for training PromptSeg.
- Extensive experiments on multiple open-source datasets demonstrate that PromptSeg outperforms existing few-shot methods in terms of segmentation accuracy and generalization capability on unseen datasets and targets, accompanied by remarkable scalability.

## 2. Related Work

### 2.1. Few-Shot Medical Image Segmentation

The few-shot segmentation method [16,17,18] learns how to generate prediction results for images (query set) containing unknown categories based on a small number of annotated examples (support set), without retraining. In few-shot learning, the most common approach is to use the “learn to learn” paradigm, constructing a learnable prototype to extract key feature representations for segmentation and transfer knowledge from the support set to the query set through feature matching. Few-shot learning methods enable models to segment query images using only a small set of examples. However, most current methods focus on a narrow range of datasets or specific tasks, which limits their generalizability and applicability across varied scenarios.

### 2.2. Prompt-Based In-Context Learning

The concept of the prompt emerges from natural language processing. Prompt-based learning directs pre-trained language models to generate desired outcomes by restructuring the problem into a specific format. GPT-3 [20] introduced the paradigm of context learning using prompts, whereby large language models can accomplish most NLP tasks through text generation by modifying prompts composed of instructions and examples. In-context learning assumes that by analyzing and emulating the examples in the prompts, LLMs can learn the implicit patterns within these examples and appropriately respond to new queries [24]. Through in-context learning, LLMs possess the potential to tackle complex tasks with just a handful of examples, including solving mathematical and logical reasoning problems. Flamingo [25] extends the capabilities of large language models to accept image and video inputs, while still utilizing language as the universal interface. With given prompts and examples, Flamingo can handle diverse vision-language tasks, such as image captioning, visual question answering, and optical character recognition (OCR).

Inpainting [22] extends the concept of prompts to the visual domain. It merges input–output image examples with the query image into a single image, generating a prediction as instructed by the prompts through image restoration. Inpainting showcases the potential of prompt-based in-context learning in tasks such as semantic segmentation, single object detection, and colorization, inspiring us to solve complex and diverse medical image segmentation tasks via visual prompts.

### 2.3. Large Vision Model

Prompt-based context learning enables models to handle different tasks based on the content of the prompts, making it possible to build a universal model to solve various problems. Concentrating on image segmentation, general segmentation algorithms for natural images have emerged by integrating massive resources and designing new proxy tasks. SAM [26] incorporates a variety of annotations, such as point and bounding box annotations, serving as prompts. This strategy enables the model to perform interactive segmentation efficiently, adapting responsively to the input prompts provided.

Painter [27] and SegGPT [28] designed a new general-purpose segmentation proxy task. They use multiple input–output pairs specified for a task as visual prompts, generating the corresponding output for query images via image inpainting. LVM [23] proposes a multi-stage training approach, which tokenizes input–output pairs and query images using VQGAN [29] and then obtains outputs by predicting the next token. These large visual models are typically designed to handle various tasks in natural images by utilizing complex proxy tasks, making them less suitable for medical imaging applications.

## 3. Methods

### 3.1. Overview of PromptSeg

A segmentation task, denoted as T:x→y, typically comprises a set of images and their corresponding pixel-wise annotations, represented as a dataset $D={\left\{\left(x_{i},y_{i}\right)\right\}}_{i=1}^{N}$. Traditional segmentation methods generally focus on training a model to fit the specific data distribution of task T, which limits the model’s applicability solely to that task.

**Figure 1:**
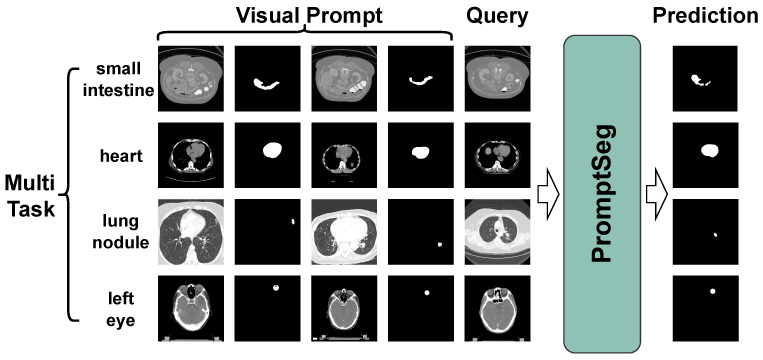
Overview of PromptSeg. Given the appropriate visual prompts, PromptSeg can solve different segmentation tasks within a single model.

As illustrated in Figure 1, we propose PromptSeg, a novel paradigm designed for generalized medical image segmentation, with a particular focus on unseen tasks. Within a probabilistic framework, PromptSeg formulates the task T as the estimation of a conditional distribution:

(1) $$p\left(y_{q}|x_{1},y_{1},\ldots ,x_{n},y_{n},q\right)≃p\left(y_{q}|q,T\right).$$

This paradigm treats a sequence of image–mask pairs $P=(x_{1},y_{1},\ldots ,x_{n},y_{n})$ specific to a task T as a visual prompt, utilizing it to guide the prediction of the target mask $y_{q}$ for a query image *q*. It is important to note that these visual prompts are not learnable parameters. Instead, they are image–mask pairs that are either provided by users or sampled from existing annotated datasets prior to inference.

From an information-theoretic perspective, we posit that the effectiveness of PromptSeg stems from uncertainty reduction, achieved by injecting task-specific information via the prompts. We formulate this process as conditional entropy minimization. In the absence of task-specific guidance, the segmentation target for a query *q* exhibits high uncertainty, quantified by the Shannon entropy $H(y_{q}|q)$. The sequence of image–mask pairs P serves as side information to mitigate this uncertainty. Our objective is to maximize the mutual information (MI) between the visual prompt P and the target $y_{q}$, which is equivalent to minimizing the conditional entropy:

(2) $$I\left(y_{q};P|q\right)=H\left(y_{q}|q\right)-H\left(y_{q}|q,P\right).$$

This formulation demonstrates that PromptSeg effectively learns to extract maximal task-specific semantics from P to eliminate the ambiguity in segmenting *q*.

**Figure 2:**
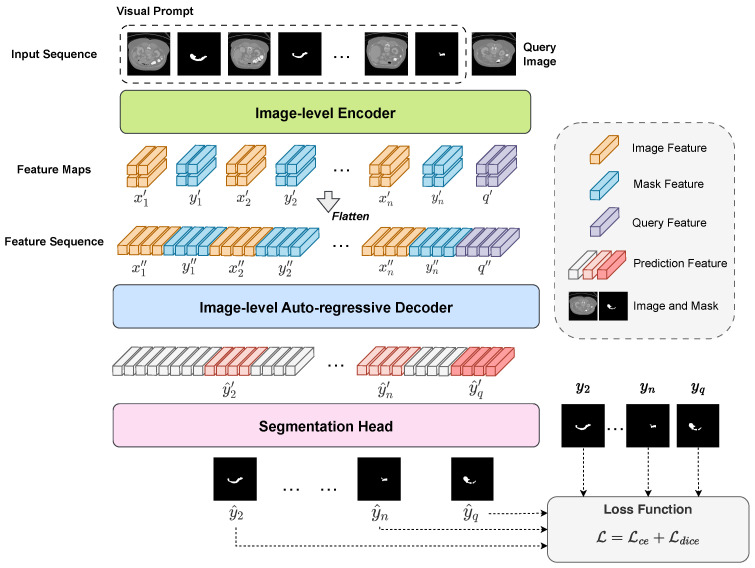
The architecture of PromptSeg. This model comprises an image-level encoder, an image-level auto-regressive decoder, and a segmentation head. First, the support visual prompts (image–mask pairs) and the query image are symmetrically processed by the ViT encoder. The extracted feature maps are then flattened and concatenated along the sequence dimension. Subsequently, the decoder utilizes an image-level causal attention mechanism to auto-regressively predict the features of target mask for the query image, strictly conditioned on the provided prompt context. Finally, a lightweight segmentation head transforms the features into a two-dimensional binary mask.

PromptSeg aims to automatically identify task patterns through contextual learning, enabling the model to segment the query image under the guidance of visual prompts. As detailed in Figure 2, PromptSeg employs a streamlined end-to-end architecture, comprising an encoder for image feature extraction and a decoder for contextual learning.

### 3.2. General Segmentation Task with Visual Prompt

Inspired by the use of prompts in natural language processing to provide instructions to models, we specify tasks through visual prompts. For a set of *n* images and their corresponding annotations $\left\{x_{\left\{1,\ldots ,n\right\}},y_{\left\{1,\ldots ,n\right\}}\right\}$ from the segmentation task T, we arrange the paired images and masks alternately to create the prompt sequence $\left[x_{1},y_{1},x_{2},y_{2},\ldots ,x_{n},y_{n}\right]$. We then input the query image *q* along with the combined input $\left[x_{1},y_{1},x_{2},y_{2},\ldots ,x_{n},y_{n},q\right]$ into the model, enabling it to refer to the sequence to perform segmentation on the query.

For the segmentation tasks $T_{\left\{1,\ldots ,K\right\}}$, we assume they contain respective target category sets $C_{\left\{1,\ldots ,K\right\}}$. If we use a single model to solve all segmentation tasks $T_{\left\{1,\ldots ,K\right\}}$, a naive implementation approach is to combine them into a multi-category segmentation task, satisfying $T^{\prime }=\cup_{i=1}^{K}T_{i}$ and containing a new category set $C^{\prime }=\cup_{i=1}^{K}C_{i}$. When the number of prompts degenerates to 0, we obtain a segmentation model containing $|C^{\prime }|$ categories. As the size of model parameters increases, models have increasingly strong fitting capabilities. Models that rely solely on the queries for segmentation fit the training set well but fail to reason from context and generalize to solve unseen tasks.

**Figure 3:**
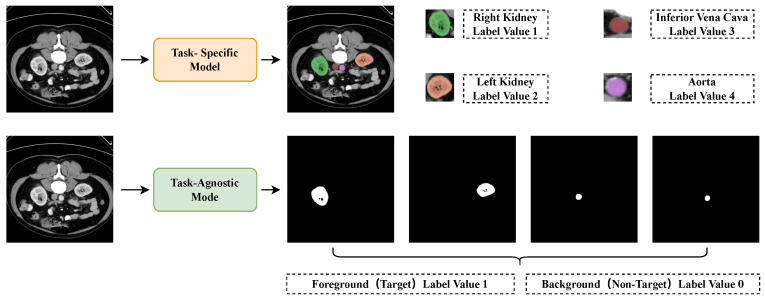
Task-specific segmentation model and task-agnostic segmentation model schemes. When using task-specific models for multi-class segmentation, each output value is strictly mapped to a predefined category, which prevents the model from extending to new tasks after training. In contrast, task-agnostic models generate binary segmentation results (foreground vs. background) and determine the specific category based on contextual information, allowing for better generalization capability to novel tasks.

To ensure that PromptSeg can recognize target features from the prompt through in-context learning, we decompose the task into binary segmentation as shown in Figure 3. Suppose that for the multi-class segmentation task $T^{\prime }$ with $|C^{\prime }|$ categories, the segmentation result of image *q* on task $T^{\prime }$ is $y_{q}\in R^{H\times W\times |C^{\prime }|}$, where H,W represent the height and width of the image. The task $T^{\prime }$ can be decomposed into $|C^{\prime }|$ binary segmentation sub-tasks $\left\{T^{1},T^{2},\ldots ,T^{|C^{\prime }|}\right\}$. Correspondingly, $y_{q}$ is decomposed into $\left\{y_{q}^{1},y_{q}^{2},\ldots ,y_{q}^{|C^{\prime }|}\right\}$. For the same query *q*, when using the prompt $P^{j}\leftarrow T^{j}$, the ground truth $y_{q}^{j}\in R^{H\times W\times 1}$ differs from the ground truth $y_{q}^{k}$ when using $P^{k}\leftarrow T^{k}$, ensuring that the model cannot directly segment based on the query but instead requires task-specific contextual information provided by the prompt to obtain the correct result.

### 3.3. Window Attention Based Encoder with Information Bottleneck

In PromptSeg, the image and mask features are extracted using an image-level encoder. To balance the computational cost and feature quality, we use a ViT-Base [30] with window attention as the encoder following SAM [26]. Specifically, at layers 2, 5, 8, and 11 of ViT-Base, 7×7 window attention is used instead of global attention to better capture local dependencies.

However, the high-dimensional features extracted by the backbone often contain redundant information (e.g., background noise) that is irrelevant to the target task. Guided by the information bottleneck (IB) principle, we aim to extract a compressed latent representation that retains sufficient task-relevant information while filtering out irrelevant nuisances.

To physically implement this, we append a lightweight bottleneck adapter after the ViT backbone. As shown in the code structure (Equation (3)), this module consists of a 1×1 convolution and a 3×3 convolution, followed by LayerNorm operations.

(3) $$Neck\left(F\right)=Conv_{3\times 3}\left(LN\left(Conv_{1\times 1}\left(F\right)\right)\right).$$

Functionally, the 1×1 convolution projects the high-dimensional ViT embedding (e.g., 768 dim) to a lower dimension (e.g., 256 dim), serving as a compressor to reduce feature redundancy. Subsequently, the 3×3 convolution aggregates local spatial context to smooth out pixel-level noise, thereby enhancing the task-relevant structural information.

The image $x_{\left\{1,\ldots ,n\right\}}$, annotations $y_{\left\{1,\ldots ,n\right\}}$, and query *q* are encoded using this shared-parameter encoder:

(4) $$\begin{array}{cc} x_{\left\{1,\ldots ,n\right\}}^{\prime } & =Neck(ViT\left(x_{\left\{1,\ldots ,n\right\}}\right)),\\ y_{\left\{1,\ldots ,n\right\}}^{\prime } & =Neck(ViT\left(y_{\left\{1,\ldots ,n\right\}}\right)),\\ q^{\prime } & =Neck(ViT(q)) \end{array}$$

The sequence of encoded feature maps $F^{\prime }=\left[x_{1}^{\prime },y_{1}^{\prime },x_{2}^{\prime },y_{2}^{\prime },\ldots ,x_{n}^{\prime },y_{n}^{\prime },q^{\prime }\right]$, where $x_{\left\{1,\ldots ,n\right\}}^{\prime }$, $y_{\left\{1,\ldots ,n\right\}}^{\prime }$ and $q^{\prime }\in R^{h\times w\times d}$, represent the compressed feature maps. Here, *d* corresponds to the bottleneck dimension, ensuring a compact and semantic-rich representation, while *h* and *w* represent the height and width of the feature map, respectively.

### 3.4. Image-Level Auto-Regressive Decoder

**Figure 4:**
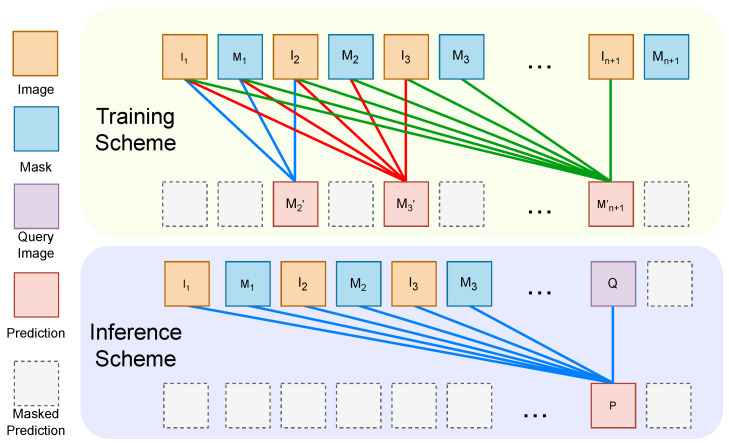
Image-level conditional probability model. (1) Training phase: the model simultaneously predicts the next image (the segmentation result) at multiple positions using visual prompts from preceding contexts. (2) Inference phase: replace any image at position ≥2 to predict the result.

As is shown in Figure 4, we design an image-level conditional probability model that extracts contextual information from the prompt in the sequence. To model the feature map sequence $F^{\prime }\in R^{(2n+1)\times h\times w\times d}$ from various images and masks, as depicted in Figure 2, we flatten it into a token sequence, $F^{\prime \prime }\in R^{S\times d}$, where

(5) $$F^{\prime \prime }=concat\left(x_{1}^{\prime \prime },y_{1}^{\prime \prime },x_{2}^{\prime \prime },y_{2}^{\prime \prime },\ldots ,x_{n}^{\prime \prime },y_{n}^{\prime \prime },q^{\prime \prime }\right)$$

where sequential features $x^{\prime \prime }$, $y^{\prime \prime }$, $q^{\prime \prime }\in R^{s\times d}$, s=h×w represents the number of tokens for each subsequence corresponding to a feature map, and *S* denotes the overall sequence length, satisfying S=h×w×(2n+1)=s×(2n+1).

To establish an image-level conditional probability model, we utilize a standard 12-layer decoder-only Transformer (structurally equivalent to the GPT-2 small [21] architecture) as our base decoder. Furthermore, we have designed image-level masked self-attention to ensure complete visibility of the preceding visual prompt for each image, while concealing the subsequent segmentation mask. This module enables the auto-regressive prediction of the mask corresponding to each image in the input sequence.

(6) $$\begin{array}{cc} {\hat{y}}_{i}^{\prime } & =Decoder(x_{i}^{\prime \prime }|x_{1}^{\prime \prime },y_{1}^{\prime \prime },\ldots ,x_{i-1}^{\prime \prime },y_{i-1}^{\prime \prime }),\\ {\hat{y}}_{i} & =SegmentationHead\left({\hat{y}}_{i}^{\prime }\right),i\in \left\{2,\ldots ,n\right\} \end{array}$$

Specifically, we designed a mechanism to mask out the subsequent information for each image that needs to be predicted in the multi-head attention.

(7) $$O=softmax(\frac{QK}{\sqrt{d}}⊙M)V$$

(8) $$M_{i,j}=\left\{\begin{array}{c} 1,\;\;\;if\;j\leq \lceil i/s\rceil *s\\ 0,\;\;\;otherwise \end{array}\right.$$

**Figure 5:**
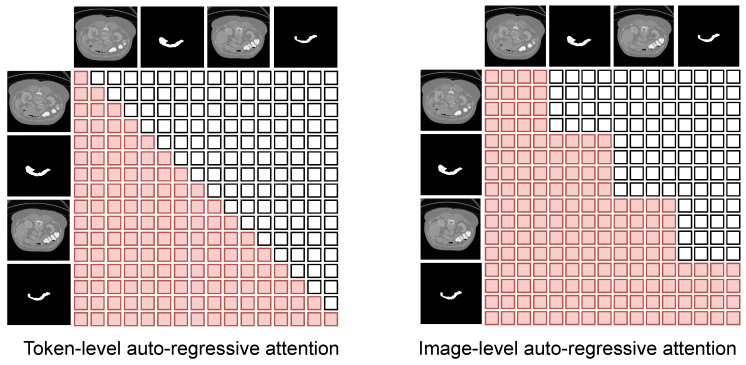
Comparison of token-level and image-level attention masks. (**Left**) Conventional token-level causal masks enforce strict token-wise autoregression, preventing tokens within the same image from attending to each other, which leads to structural fragmentation. (**Right**) Our image-level autoregressive mask. Spatial tokens within the same query or prompt image are fully visible to each other (see unmasked diagonal blocks). This design ensures holistic spatial coherency and structural continuity.

As is shown in Figure 5, each token can perceive other tokens within the same image by employing an image-level mask instead of a token-level mask, ensuring the coherence of the features.

Figure 4 shows the training and inference schemes of PromptSeg. To ensure feature consistency and maximize training efficiency, the prompt images and the target query image share an identical encoding process. During the training phase, we utilize n+1 image–mask pairs as input to enable multi-position parallel training. In this next-image prediction process, the first *k* image–mask pairs serve as a *k*-pair visual prompt for the subsequent image $x_{k+1}$ (1≤k≤n). Thus, the input prompt images inherently act as intermediate queries themselves. The outputs corresponding to $x_{\left\{2,3,\ldots ,n+1\right\}}$ are supervised and denoted as ${\hat{y}}_{\left\{2,3,\ldots ,n+1\right\}}$, while outputs from other positions are deprecated. The loss function is defined as:

(9) $$L_{total}=\frac{1}{n}\sum_{i=2}^{n+1}L\left({\hat{y}}_{i},y_{i}\right)$$

During the inference process, PromptSeg can handle a variable number of prompt pairs, ranging from 1 to *n* prompt pairs, followed by a query image. The loss used for training our PromptSeg is defined as a combination of distribution-based and region-based objectives: $L=L_{ce}+L_{dice}$.

The cross-entropy loss $L_{ce}$ is employed to measure the pixel-wise classification accuracy by minimizing the divergence between the predicted probability distribution and the ground truth. It is formulated as:

(10) $$L_{ce}=-\frac{1}{N}\sum_{i=1}^{N}\left[y_{i}\log\left(p_{i}\right)+\left(1-y_{i}\right)\log\left(1-p_{i}\right)\right]$$

where *N* denotes the total number of pixels in the query image, $y_{i}\in \left\{0,1\right\}$ represents the ground truth label of pixel *i*, and $p_{i}\in \left[0,1\right]$ is the predicted probability of the foreground class. From an information-theoretic perspective, optimizing the network via the cross-entropy loss in Equation (10) is equivalent to minimizing the empirical conditional entropy defined in Equation (2). By injecting the visual prompt P as a conditioning context, the model extracts task-specific semantics to reduce the uncertainty of the query. This results in a sharper predicted probability distribution $p_{i}$, which directly minimizes $L_{ce}$ and guides the parameter updates to better utilize the prompt.

To mitigate the issue of class imbalance, where the anatomical target often occupies a small portion of the scan, we incorporate the Dice loss $L_{dice}$. This loss directly optimizes the structural overlap between the prediction and the target mask:

(11) $$L_{dice}=1-\frac{2\sum_{i=1}^{N}y_{i}p_{i}+\epsilon }{\sum_{i=1}^{N}y_{i}+\sum_{i=1}^{N}p_{i}+\epsilon }$$

where ϵ is a smooth term (set to 1 × 10^−5^) used to prevent division by zero and improve numerical stability.

## 4. Experiments

### 4.1. Dataset and Metrics

We collect 20 open-source and 4 private medical segmentation datasets for training and analyzing PromptSeg’s general segmentation capabilities, involving different data modalities, anatomical structures, and segmentation targets. Among them, four datasets, SegThor [31], BTCV [32], CHAOS [33], and Abdominal-OAR, are fully held out for evaluating the effectiveness of various methods and do not participate in the train-validation process. SegThor and BTCV are public CT datasets used for the segmentation of organs-at-risk (OAR) in the thoracic and abdominopelvic regions, respectively. CHAOS is a public MRI dataset reserved for evaluating cross-modality performance. Abdominal-OAR is a private OAR segmentation dataset for liver cancer, collected by the Radiotherapy Department of Anhui Provincial Hospital, where all CT images were annotated by two experienced physicists. In addition, we retain four unique segmentation targets from the TotalSegmentatorV2 [34] dataset—lung upper/lower lobe right/left (Lung-ulr, Lung-llr, Lung-ull, and Lung-lll)—which do not appear in other datasets, for evaluation as unseen targets. This implies that slices containing these classes are excluded from the training process. All MRI datasets utilize the T1 modality. Each dataset involved in the train-validation process is divided into a training validation set at a proportion of 8:2. The detailed information of datasets is listed in Table 1.

All 3D scans are reformatted into 2D axial slices, with the data annotations similarly structured into 2D masks. Separate binary masks are generated for each class within the same 2D slice.

We utilize the Dice similarity coefficient (DSC) as the evaluation metric. For datasets with multiple segmentation targets, we calculate the average performance across all classes. To avoid the random effect of the low-quality prompts, we randomly generate five different prompt sequences for all query images during evaluation and use the average DSC as the final result. In practice, a low-quality prompt typically refers to a sub-optimal slice situated at organ boundaries, corrupted by imaging artifacts, or lacking representative anatomical features.

### 4.2. Implementation Details

We use PyTorch-2.2.0 to implement the proposed method. All models are trained from scratch using 8 NVIDIA H800 GPUs with 80 GB memory. For data preprocessing, distinct strategies are applied to align with the physical properties of different modalities. CT images are clipped according to the specific window width and level corresponding to the segmentation target, followed by min-max normalization. In contrast, MRI images, lacking standardized intensity values, are clipped at 0.5% and 99.5% of each sample’s intensity histogram before performing min-max normalization to mitigate outlier effects.

The resolution of each input image is 256 × 256, and the patch size is 16 × 16. For each query image, we generate the input sequence or support set by sampling *n* pairs of image–mask with identical category from the corresponding dataset. In the comparative experiments, *n* is set to 7, which equals a 7-shot setting in few-shot learning methods. After each training epoch, the input sequences are resampled to generate new prompt-query sequences. The support set is also resampled in the same way.

The model configuration of PromptSeg comprises an encoder and a decoder, each constructed from 12 Transformer layers. The batch size is set to 2, implying that in the case of *n* equals 7, each training step will concurrently process 32 images on each GPU. During training, we employ an AdamW optimizer with a cosine learning rate scheduler. All methods employ the same data augmentation approach, namely selecting an augmentation method at random from those methods shown in Table 2 each time, and performing data augmentation with the same parameters on the query image and its visual prompts with a probability of p=0.5. The other training hyperparameters include a base learning rate of 2 × 10^−4^, a weight decay of 0.01, $\beta_{1}$ set to 0.9, $\beta_{2}$ set to 0.999, and a one-epoch warm-up period.

The comparative experiments are conducted in two phases to evaluate performance and generalization. First, for the CT modality, models are trained from scratch for five epochs using the collected datasets, explicitly excluding CHAOS, AMOS-MRI, and TotalSegmentator-MRI. Second, for the MRI modality, we perform cross-modality fine-tuning. Models pre-trained on CT are fine-tuned on AMOS-MRI and TotalSegmentator-MRI for approximately 5000 steps, with the learning rate reduced to half of the first phase.

### 4.3. Comparative Experiments on CT Modality

We compare the proposed PromptSeg with other existing methods including RPT [18], CATNet [17], ALPNet [16], and UniverSeg [19]. Among them, RPT, CATNet, and ALPNet are typical few-shot learning methods, and UniverSeg is the state-of-the-art (SOTA) general medical segmentation model before PromptSeg. It is crucial to emphasize that during the evaluation on these unseen datasets and targets, the model parameters were strictly frozen. The model relies entirely on the provided visual prompts or support set for task adaptation, achieving true training-free transfer.

To evaluate the universality of PromptSeg on the dominant modality in medical imaging, we first conduct extensive comparisons on unseen CT datasets. Experiment results reported in Table 3 and Table 4 demonstrate that PromptSeg performs well on all three unseen datasets and four unseen targets. On the datasets never seen during training, our PromptSeg achieves 55.72% DSC on SegThor, 63.57% DSC on BTCV, and 84.70% DSC on Abdominal-OAR, at least outperforming the other methods by 0.41%, 9.31%, and 9.11%, respectively. On the four classes of unseen segmentation targets, our PromptSeg has achieved 73.82% DSC, 65.17% DSC, and 63.65% DSC on Lung-ulr, Lung-llr, and Lung-lll, respectively, surpassing the other approaches by a bare minimum of 3.52%, 1.81%, and 4.93%, respectively. For the Lung-ull class, we obtain a DSC of 63.35%, only 0.84% lower than the best-performing model ALPNet. The consistent superiority of our method relative to existing methods on these tasks demonstrates the outstanding generalization ability and effectiveness of PromptSeg.

### 4.4. Comparative Experiments on MRI Modality

An MRI image presents different imaging characteristics compared to CT, relying on proton density and magnetic properties rather than electron density (Hounsfield units). Furthermore, publicly available annotated MRI datasets are significantly scarcer than CT datasets, posing challenges for training large-scale universal models from scratch. To address these challenges, we extended our evaluation to the MRI modality by leveraging the representations learned from the extensive CT data. We initialized PromptSeg with CT-pretrained weights and performed minimal adaptation on the MRI support sets. We compared PromptSeg with the same baselines (RPT, CATNet, ALPNet, and UniverSeg) using the CHAOS dataset as a held-out test set.

As summarized in Table 5, PromptSeg demonstrates superior overall performance in this transfer setting, achieving the highest average DSC of 70.24%. This outperforms the state-of-the-art universal model, UniverSeg (68.73%), and significantly surpasses the best few-shot baseline, ALPNet (63.56%). Specifically, PromptSeg exhibits remarkable robustness on major organs, achieving 83.94% DSC on the liver and an average of 68.70% on the kidneys, surpassing UniverSeg by substantial margins. However, a performance drop is observed on the spleen target (59.67%). This can be attributed to the negative transfer of strong priors learned from CT data. In CT images, the spleen typically presents as an organ with intensity similar to or higher than the liver. Conversely, in T1-weighted MRI, the spleen appears hypointense (darker than the liver). This intensity inversion between modalities conflicts with the strong feature priors captured by PromptSeg during its extensive CT training, leading to misinterpretation in the MRI domain. Despite this specific challenge, PromptSeg maintains the best overall performance, confirming that for most anatomical structures, the structural semantics learned from CT can be effectively transferred to MRI via visual prompts.

This experiment validates PromptSeg’s capability for robust in-context learning across modalities. Despite the intensity inversion challenge in the spleen, the model effectively extracts contextual structural semantics from visual prompts to guide the segmentation. It suggests that the information bottleneck encoder minimizes conditional entropy by filtering modality-specific noise, enabling the model to generalize based on invariant shapes rather than low-level textures.

## 5. Discussion

While the experimental results have demonstrated the superior performance and cross-modality generalization of PromptSeg, the underlying working mechanism remains a critical subject of inquiry.

In this section, we first conduct an ablation study to isolate the contribution of each core architectural component. Subsequently, we examine the model’s fidelity in following visual prompts. This validates the foundational reliability of the prompt-based interaction. Then, we explore the scalability of the framework by evaluating its performance under varying training and inference settings. This analysis highlights the model’s inference flexibility and deployment efficiency, demonstrating its adaptability to different computational constraints. Next, to provide an interpretable perspective on these behaviors, we visualize and quantify the predictive uncertainty using Shannon entropy. This metric serves as a proxy for the model’s confidence, revealing how visual prompts as side information effectively reduce the uncertainty of the prediction and verifying the underlying mechanism of the proposed framework. Finally, we discuss the inherent limitations and trade-offs of PromptSeg to provide a comprehensive view of its clinical applicability and guide future research.

### 5.1. Ablation Study

To explicitly isolate and quantify the contribution of the core components in our framework, we conducted comprehensive ablation studies evaluated across four CT datasets.

**Prompt Mechanism.** Within our task-agnostic framework, multi-class tasks are uniformly decomposed into binary sub-tasks (foreground vs. background). Consequently, conducting an ablation with absolutely no visual prompts (n=0) is mathematically ill-posed, as the segmentation target becomes entirely undefined without task-specific context. Instead, we utilize the 1-shot setting (n=1) as the extreme minimal conditioning baseline. As will be demonstrated in the subsequent scaling study (Section 5.3), minimal prompting yields highly constrained performance. The substantial performance surge from n=1 to n=7 explicitly verifies the prompt mechanism as the foundational driver of ambiguity resolution.

**Architectural Components.** Furthermore, we ablated two critical architectural designs under the fixed 7-shot setting. The quantitative results are summarized in Table 6.

As shown in the first row of Table 6, removing the bottleneck adapter (comprising the 1 × 1 and 3 × 3 convolutions in Equation (3)) and routing raw high-dimensional ViT features directly to the decoder causes a precipitous performance decline. Notably, the DSC scores plummeted from 55.72% to 28.66% on SegThor and from 66.50% to 57.51% on TotalSegmentator. These results underscore the bottleneck adapter’s indispensable role in filtering noise and compressing redundant information, serving as a concrete physical implementation of our information bottleneck (IB) principle.

Subsequently, we substituted our proposed image-level mask with a conventional token-level auto-regressive mask. This alteration led to a significant performance degradation, most notably a margin of over 5% (from 66.50% to 61.37%) on the challenging TotalSegmentator dataset. Unlike natural language processing (NLP), where unidirectional causality is inherent to sequence modeling, dense spatial features in medical imaging necessitate bidirectional context within each individual image to resolve local ambiguities. These results confirm that image-level visibility is indispensable for preserving spatial coherency and structural continuity during dense prediction tasks.

### 5.2. Prompt Following Study

**Figure 6:**
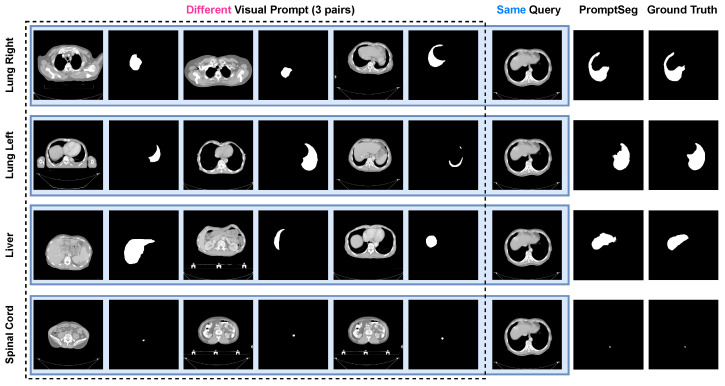
Segmentation results of same query image with different visual prompts. Upon provision of prompts with diverse segmentation targets, PromptSeg generates disparate segmentation results for an identical query image.

PromptSeg is proposed to identify task patterns through contextual information in visual prompts and guide the model to segment specified targets from query images. When visual prompts with different segmentation targets are provided for the same query image, PromptSeg can automatically identify the types of segmentation targets based on the visual prompts and generate corresponding segmentation results. As shown in Figure 6, we provide PromptSeg with four sets of visual prompts, each targeting a different segmentation: right lung, left lung, liver, and spine cord. Each set of visual prompts ends with an identical query image, encompassing all four segmentation targets. It is evident that PromptSeg completely follows the visual prompts, exclusively generating segmentation prediction outcomes that coincide with the present visual prompts in every inference. This indicates that PromptSeg has a good ability to follow prompts and can understand prompts well.

### 5.3. Scaling Study

**Figure 7:**
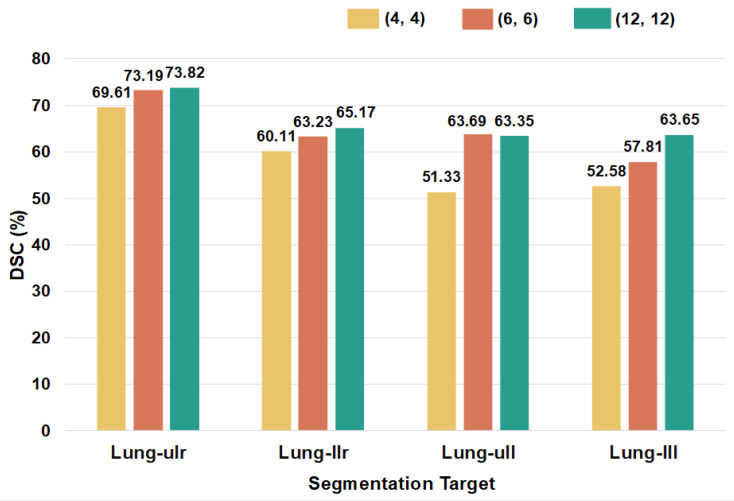
Segmentation performance of diverse model sizes on the four unseen segmentation targets. The legend (4,4) denotes a model architecture wherein the model’s encoder and decoder each possess 4 Transformer layers.

We conduct training on PromptSeg with varying parameter quantities to explore its scalability on the model side. Specifically, the encoder of PromptSeg expanded from four Transformer layers to twelve, with the decoder correspondingly possessing an equivalent number of layers as the encoder. As shown in Figure 7, the performance of these models on four unseen targets suggests that our model’s generalization capabilities are enhanced concurrently with the scaling of parameters.

Furthermore, we train PromptSeg with varying prompt sequence length configurations to examine its scalability efficacy on the visual prompt side. As shown in Table 7, when the number of image–mask pairs provided to train the model as visual prompts gradually increases from 1 to 11, the segmentation accuracy of PromptSeg also increases accordingly on different datasets. Benefiting from the sequential design, image-level auto-regressive decoder, and the loss elucidated in (9), when trained with n pairs of visual prompts, PromptSeg can accommodate an equal or lesser number of visual prompt pairs during inference. We further conduct scaling experiments on PromptSeg during the inference stage. For the model trained with n=7, we provide three to seven pairs of visual prompts during inference. The results presented in Table 8 illustrate that across all four test datasets, the segmentation outcomes consistently demonstrate a characteristic of growing with the increase in the number of input visual prompts. In comparison with Table 7, it becomes apparent that the model trained under the setting of n=7 exhibits comparable performance to a model specifically trained under the setting of n=3, when the quantity of visual prompt pairs is set at three. This signifies that we can accommodate a variety of visual prompt number input scenarios by training a model with a larger *n*, which is quite effective for practical applications and deployments.

In conjunction with the prompt following study and scaling study, we suggest that the segmentation ability of PromptSeg on unseen tasks stems from its understanding and following of visual prompts, and amplifies with the increase of model parameters and provided visual prompts.

### 5.4. Entropy Dynamics and Uncertainty Quantification

To provide a theoretical interpretation of the model’s behavior, we visualize and quantify the predictive uncertainty using Shannon entropy. We employ a model trained with a fixed 7-shot setting (n=7) and evaluate it under varying inference settings to observe the dynamic evolution of uncertainty.

In our binary segmentation task, the model output passes through a Softmax layer to produce a probability map. Let $p_{i,c}\in \left[0,1\right]$ denote the predicted probability of class *c* at pixel *i*, where C=2 corresponds to the background (c=0) and the foreground target (c=1). Since $\sum_{c=0}^{1}p_{i,c}=1$, the pixel-wise entropy $H_{i}$ is calculated by summing the contributions of both classes:

(12) $$H_{i}=-\sum_{c=0}^{1}p_{i,c}\log\left(p_{i,c}+\epsilon \right)$$

where $\epsilon =10^{-8}$ is a small constant for numerical stability. A higher $H_{i}$ indicates greater uncertainty in the model’s prediction at that location, whereas a lower value implies high confidence in either the background or the foreground.

**Figure 8:**
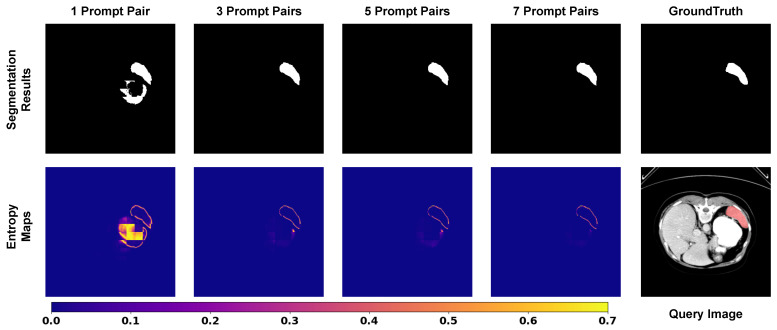
Visualization of entropy dynamics. In the 1-shot setting, high uncertainty (red/yellow) dominates the prediction. As prompts increase, the uncertainty is effectively minimized, remaining only at the object boundaries.

We first visualize the pixel-wise entropy maps in Figure 8 to understand how visual contexts influence decision confidence. In the 1-shot setting, we observe that the contour of the segmentation target (e.g., spleen) exhibits high entropy. This aligns with the expectation in medical image segmentation, where boundaries are inherently ambiguous. However, critically, we also observe substantial high-entropy clusters in the adjacent non-target background. These regions correspond to false positive predictions (artifacts), indicating high epistemic uncertainty where the limited context fails to distinguish the foreground from similar surrounding tissues. As the number of prompt pairs increases, a clear trend emerges. The transition from 1-shot to 3-shot yields a significant reduction in background artifacts, and by the 7-shot setting, these non-target high-entropy regions effectively vanish. Simultaneously, the entropy values at the boundaries show a consistent downward trend. This suggests that the model integrates richer contextual representations from the expanded support set, gaining more information to resolve local ambiguities. This visualization confirms that increasing the number of prompts effectively transforms the model’s state from high uncertainty to high confidence, specifically by suppressing the ambiguity in non-target regions.

Complementing the visualization, we perform a quantitative evaluation on the BTCV dataset to measure the global reduction in uncertainty. We calculate the average pixel-wise entropy across the test set under different prompt settings. As presented in Table 9, the average entropy exhibits a monotonic decrease as the number of prompt pairs increases. Specifically, the sharpest decline occurs when transitioning from the 1-shot to the 3-shot setting, which aligns perfectly with the rapid performance gain observed in the segmentation metrics. Subsequent additions (from three to seven pairs) yield smaller marginal reductions in entropy. This trajectory is consistent with the segmentation performance in Table 7 and Table 8, validating that PromptSeg optimizes segmentation performance by explicitly minimizing the conditional entropy of the target distribution, thereby ensuring reliable decision making in complex medical scenarios.

### 5.5. Limitations

While PromptSeg demonstrates strong few-shot generalization capabilities, it possesses several inherent limitations that must be acknowledged for practical clinical deployment.

**Two-Dimensional Slice-by-Slice Processing.** First and foremost, although clinical CT and MRI data are volumetric, PromptSeg is fundamentally a 2D segmentation framework. It processes 3D volumes in a slice-by-slice manner. While this design significantly reduces the GPU memory footprint required for in-context sequence reasoning, it inherently discards critical inter-slice spatial context along the Z-axis, which can sometimes lead to disjointed predictions across continuous anatomical structures.

**Computational Trade-offs for Multi-Target Parsing.** A primary engineering limitation stems from the conditional binary decomposition. To segment *K* distinct anatomical structures simultaneously, the framework requires *K* independent forward passes. To quantify this overhead, we profiled the computational complexity and inference efficiency on a standard commercial GPU, as summarized in Table 10.

As demonstrated, even under the 7-shot setting, PromptSeg achieves an inference latency of 42.90 ms per slice (∼23.3 FPS) with a highly affordable memory footprint of ∼3.4 GB. While this efficiency is highly practical and acceptable for on-demand, interactive clinical queries, the linear scaling of computational cost for comprehensive whole-body parsing leaves substantial room for further algorithmic acceleration.

**Dataset Construction and Evaluation Protocols.** Regarding dataset construction, our current training corpus is predominantly composed of CT scans, which inherently limits the comprehensive verification of cross-modality capabilities. Furthermore, while our framework demonstrates strong generalization on unseen targets and cross-modality datasets, evaluating universal generalization through a strictly isolated cross-region paradigm (e.g., training exclusively on abdominal organs and testing on thoracic organs) was not conducted. Such rigorous cross-region evaluations will be a primary focus in our future scaled-up iterations.

**Figure 9:**
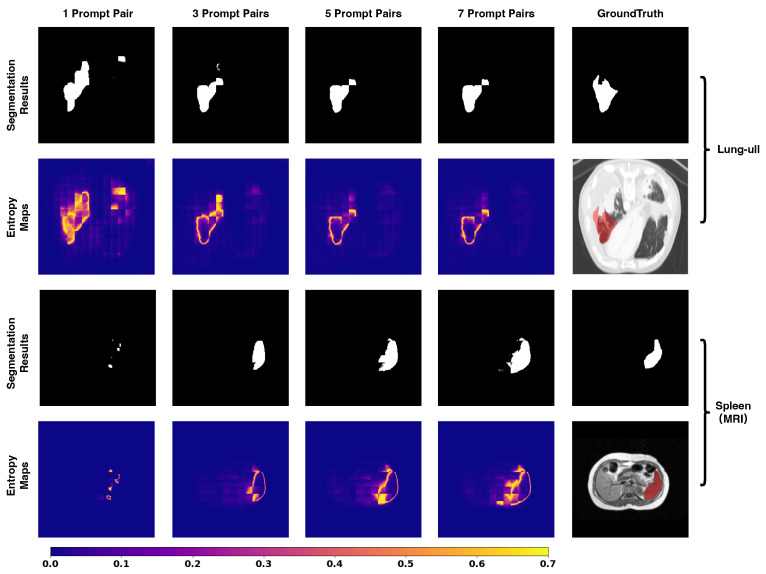
Visual results for lung lobe (CT) and spleen (MRI) segmentation. While the segmentation performance steadily improves with an increasing number of prompts, the prediction maps still exhibit high entropy values.

**Performance on Complex Tasks and Modality Inversion.** Moreover, PromptSeg currently exhibits sub-optimal performance when confronted with highly complex anatomical topologies and severe modality inversion. As visualized in Figure 9, in the challenging tasks of the left upper lung lobe (CT-Lung-ull) and the spleen under MRI modality, the segmentation results intuitively improve as the number of visual prompts increases. However, the corresponding entropy maps indicate that the model’s predictive uncertainty remains relatively high in the boundary regions. Consequently, the segmentation precision in these extreme corner cases has not yet met the rigorous standards required for direct clinical application. Enhancing the model’s robustness, entropy reduction efficiency, and accuracy on such difficult tasks remains a critical objective for future development.

## 6. Conclusions

In this work, we propose PromptSeg, an end-to-end universal medical image segmentation method based on prompt learning. We introduce the approach of prompt learning into medical image segmentation, enabling the model to generalize to unseen tasks without additional training by providing a handful of task-specific visual prompts. Our extensive experiments on multiple held-out datasets demonstrate that PromptSeg outperforms existing state-of-the-art few-shot and universal methods on almost all unseen targets. Notably, the model exhibits strong cross-modality robustness, successfully transferring structural knowledge from CT to MRI for major organs, proving the versatility of the proposed framework.

Beyond standard performance metrics, we provide a theoretical interpretation of the model’s effectiveness. Through the visualization of entropy dynamics and quantitative uncertainty analysis, we demonstrate that the model’s generalization capability stems from the minimization of predictive uncertainty via visual contexts. The visual prompts serve as effective side information, collapsing the hypothesis space and suppressing the uncertainty in the query image.

Furthermore, our study highlights the remarkable scalability and inference efficiency of PromptSeg. The model supports flexible deployment with varying numbers of prompts, allowing users to balance computational cost and segmentation precision in real-world clinical scenarios. In the future, we aim to expand the scale of PromptSeg by integrating modern large vision models as the visual backbone to further enhance feature representation. Additionally, we plan to address the specific challenges of negative transfer observed in cross-modality tasks (e.g., intensity inversion in the spleen) by developing lightweight, modality-specific adapters to decouple texture representation from structural priors, ultimately maximizing the model’s universality across diverse imaging protocols.

## Figures and Tables

**Table 1:** Dataset information.

Dataset	Modality	Samples	Classes	Train	Valid	Test
Public Datasets						
TotalSegmentatorV2 [34]	CT	1228	113	982	246	∖
TotalSegmentatorV2 [34]	CT	1057	4	∖	∖	1057
TotalSegmentatorMRI (Abdominal) [35]	MRI	51	13	41	10	∖
CHAOS [33]	MRI	20	4	∖	∖	20
MSD Heart [36]	CT	20	1	16	4	∖
MSD Lung [36]	CT	63	1	50	13	∖
MSD Pancreas [36]	CT	281	2	224	57	∖
MSD HepaticVessel [36]	MRI	303	2	242	61	∖
MSD Spleen [36]	CT	41	1	32	9	∖
MSD Colon [36]	CT	126	1	100	26	∖
VerSe [37]	CT	374	28	300	74	∖
AMOS-CT [38]	CT	300	15	240	60	∖
AMOS-MRI [38]	MRI	60	13	48	12	∖
LIDC [39]	CT	726	1	580	146	∖
StructSeg Thor [40]	CT	40	6	32	8	∖
StructSeg HaN [40]	CT	40	22	32	8	∖
StructSeg ThorGTV [40]	CT	40	1	32	8	∖
StructSeg HaNGTV [40]	CT∖MRI	40	1	32	8	∖
Covid-Seg [41]	CT	199	1	159	40	∖
SegThor [31]	CT	40	4	∖	∖	40
BTCV [32]	CT	30	13	∖	∖	30
Private Datasets						
Abdominal OAR	CT	39	8	∖	∖	39
Stomach OAR	CT	36	9	28	8	∖
Lung OAR	CT	90	4	72	18	∖
Lung Tumor	CT	80	1	64	16	∖

**Table 2:** Data augmentation methods.

Augmentation	Description
Flip Intensities	1−image for all images (query and prompts)
Flip Labels	1−mask for all masks
Horizontal Flip	Flip all images and masks horizontally
Vertical Flip	Flip all images and masks vertically
Sobel-Edge Label	Apply a Sobel filter to each mask
Affine Shift	Affine shift for all images and masks
Brightness Contrast Change	Change brightness and contrast for all images
Elastic Warp	Elastic deformable warp for all images and masks
Gaussian Blur	Add Gaussian blur for all images
Gaussian Noise	Add Gaussian noise for all images
Sharpness Change	Change sharpness for all images

**Table 3:** Comparison with other methods in Dice similarity coefficient (%, mean ± std) on the held-out CT datasets. Best results are highlighted in **bold**.

Method	Unseen Datasets		
	SegThor	BTCV	Abdominal OAR
Few-Shot Model			
ALPNet [16]	36.39 ± 0.08	44.49 ± 0.10	74.09 ± 0.05
CATNet [17]	22.59 ± 0.22	24.88 ± 0.17	43.60 ± 0.07
RPT [18]	32.84 ± 0.11	50.05 ± 0.32	75.59 ± 0.13
Universal Model			
UniverSeg [19]	55.31 ± 0.53	54.26 ± 0.37	74.97 ± 0.87
**PromptSeg**	**55.72 ± 0.28**	**63.57 ± 0.25**	**84.70 ± 0.41**

**Table 4:** Comparison with other methods in Dice similarity coefficient (%, mean ± std) on the retained four unseen targets. Best results are highlighted in **bold**.

Method	Unseen Targets			
	Lung-ulr	Lung-llr	Lung-ull	Lung-lll
Few-Shot Model				
ALPNet [16]	70.30 ± 1.25	63.36 ± 0.11	**64.19 ± 0.52**	57.43 ± 0.50
CATNet [17]	26.33 ± 1.02	20.16 ± 0.66	16.85 ± 0.75	15.55 ± 0.70
RPT [18]	63.06 ± 0.54	55.98 ± 0.19	57.95 ± 0.47	52.10 ± 0.35
Universal Model				
UniverSeg [19]	63.31 ± 0.20	63.21 ± 0.35	60.67 ± 0.22	58.72 ± 0.29
**PromptSeg**	**73.82 ± 0.45**	**65.17 ± 0.24**	63.35 ± 0.25	**63.65 ± 0.09**

**Table 5:** Cross-modality generalization comparison on the unseen CHAOS MRI dataset in Dice similarity coefficient (%, mean ± std). Best results are highlighted in **bold**.

Method	Segmentation Targets (CHAOS)				Average
	Liver	Right Kidney	Spleen	Left Kidney	
Few-Shot Model					
ALPNet [16]	78.98 ± 0.53	56.41 ± 0.36	59.74 ± 0.92	59.10 ± 0.65	63.56 ± 0.33
CATNet [17]	50.43 ± 0.09	13.13 ± 0.32	15.40 ± 0.13	12.63 ± 0.12	22.90 ± 0.07
RPT [18]	59.83 ± 0.17	62.81 ± 1.54	21.53 ± 0.43	54.61 ± 5.00	49.70 ± 1.38
Universal Model					
UniverSeg [19]	73.69 ± 0.82	68.04 ± 1.20	**62.62 ± 1.17**	**70.53 ± 1.23**	68.73 ± 0.35
**PromptSeg**	**83.94 ± 0.96**	**73.48 ± 1.63**	59.67 ± 1.65	63.86 ± 2.39	**70.24 ± 0.81**

**Table 6:** Ablation study of architectural components (evaluated under 7-shot setting using DSC %, and best results are highlighted in **bold**).

Model Variant	Abdominal OAR	SegThor [31]	BTCV [32]	TotalSeg- mentatorV2 [34]
w/o Bottleneck Adapter	72.33	28.66	48.63	57.51
w/ Token-level Mask	82.38	54.99	63.27	61.37
PromptSeg	**84.70**	**55.72**	**63.57**	**66.50**

**Table 7:** Segmentation performance of the same model trained with different prompt pairs. Best results are highlighted in **bold**.

Pairs	SegThor [31]	BTCV [32]	Abdominal OAR	TotalSeg- mentatorV2 [34]
1	51.25	58.05	80.29	13.50
3	53.65	63.26	83.02	46.40
7	55.72	63.57	84.70	**66.50**
11	**61.29**	**66.54**	**86.58**	65.41

**Table 8:** Segmentation performance scaling with varying numbers of visual prompts during inference. The model is trained with a fixed 7-shot setting (n=7), demonstrating inference flexibility. Best results are highlighted in **bold**.

Pairs	SegThor [31]	BTCV [32]	Abdominal OAR	TotalSeg- mentatorV2 [34]
3	53.40	61.72	83.34	59.70
4	54.29	62.55	84.11	62.60
5	54.86	63.31	84.37	64.37
6	55.36	63.47	84.64	65.60
7	**55.72**	**63.57**	**84.70**	**66.50**

**Table 9:** Quantitative analysis of predictive uncertainty on the BTCV dataset. The average pixel-wise entropy decreases monotonically as more visual prompts are provided, indicating higher model confidence. (↓: lower is better).

Number of Prompts	1	3	5	7
Average Entropy (↓)	0.008435	0.005974	0.005682	0.005652

**Table 10:** Computational complexity and inference efficiency of PromptSeg across different numbers of visual prompts (*n*).

Prompts (*n*)	Params (M)	FLOPs (G)	Memory (MB)	Inference (ms/Slice)
1	358.90	218.83	2219.00	13.83
3	358.90	714.48	2428.14	18.22
7	358.90	1937.69	3452.65	42.90

## Data Availability

Data are contained within the article.
